# The Concours in France

**Published:** 1847-12

**Authors:** 


*The concours in France.*—In the Journal des Connaissances Medico-Chirurgicales for July, there is a full report of the discussion in the Chamber of Peers on the bill for regulating medical practice in France. During its progress M. M. Cousin and Flourens offered an amendment by which the Concours for the places of Professors in the Schools of Medicine is abolished, and the following plan substituted : Each of the following bodies, viz : the Faculty in which the vacancy occurs, the Academy of Sciences and the Academy of Medicine, presents the names of two candidates, from among whom the minister of public instruction selects one to fill the vacant chair. This plan has been long pursued at the College of France and Museum of Natural History, and was formerly in vogue in the School of Medicine, and M. Cousin says it has always worked well. The amendment was adopted by the Peers.

It has been stated by a correspondent of the Annalist that this abolition of the concours is a government measure, brought forward to give it more patronage. This is an error. The law proposed by the minister and reported by the committee, retained the concours, and it was at the suggestion of the distinguished men named above, and by their efforts, supported by those of M. Thenard, that the amendment was adopted.

The concours is retained for all places except those of the highest degrees of instruction, i. e., professors in the schools which have the power of conferring the degree of doctor of medicine. The arguments of M. M. Cousin & Thenard, were very able, showing that the concours was inconvenient, consumed a great part of the time of the professors, was harrassing to the candidates, and often repelled, from this circumstance, men of the highest talents and qualifications, who would forego the chance of obtaining the highest places rather than submit to the annoyance, vexation and mortification of a concours. Many examples in point were related, among others Louis, Magendie & Broussais—the latter obtained his chair by appointment, the two former are not professors in the School of Medicine. None of the three have ever concoured.—*Southern Jour. Med. and Pharmacy.*

